# The Impact of the Drug Epidemic on the Incidence of Sepsis in West Virginia

**DOI:** 10.7759/cureus.3521

**Published:** 2018-10-30

**Authors:** Frank H Annie, Mark C Bates, Chris K Uejio, Abhishek Bhagat, Tanureet Kochar, Sarah Embrey

**Affiliations:** 1 Cardiology, Charleston Area Medical Center, Charleston, USA; 2 Miscellaneous, Florida State University, Tallahassee, USA; 3 Internal Medicine, Charleston Area Medical Center, Charleston, USA; 4 Pharmacy, University of Charleston School of Pharmacy, Charleston, USA

**Keywords:** sepsis, drug use, intravenous drug use

## Abstract

Introduction

Drug abuse and overdoses are on the rise in West Virginia. Multiple socioeconomic and prescription-prescribing practices influenced this shift. The shifting burden of intravenous drug use to more rural areas has created unique challenges for patient access (medical attention, addiction education, rehabilitation), as well as created an avalanche of additional costs for hospital networks.

Methods

We analyzed sepsis cases from 2006 to 2015 to investigate whether different types of drug use have increased the odds of developing sepsis as compared to other forms of drug use. To investigate this aspect, the authors examined this relationship by using a logistical regression and a time series analysis of the total cases of drug use and infections.

Results

The initial analysis investigated the association between drug use and the number of sepsis cases at Charleston Area Medical Center from 2006 to 2015 using a time series analysis. Results suggest that there are similar relationships between sepsis and sedative usage (p=0.016) and sepsis by mixed/other drug (p= 0.020) use. For logistic regression (n=2284), the infection models of sepsis/skin, endocarditis/skin infection, and osteomyelitis/skin infection showed several exposures significantly increased the risk of different infections. A drug user with a positive urine test for opiates is 80.8 percent more likely to develop sepsis as compared to skin infections (p=0.001). The use of sedatives also significantly increased the odds of developing sepsis by 83.2 percent (p=0.002).

Conclusion

Sepsis left untreated will result in a high mortality rate. As illicit drug use increases, sepsis cases will increase. Further research is needed to understand the continued relationship between drug use and the incidence of sepsis. Based on the current evidence, sepsis appears to be slightly affected by drug use and seems to be influenced by sedatives and opiates but only at a marginal level.

## Introduction

Intravenous drug use (IVDU), which increases the risk of skin and blood infections, correspondingly increased in the study area. The transmission of blood-borne diseases, such as human immunodeficiency virus (HIV) and hepatitis C have been used as an indicator of increased drug use [1]. Widespread intravenous drug misuse caused a surge of these illnesses, a dramatic loss of productivity, reduced the long-term quality of life, and increased health care costs [2-4].

Sepsis incidence can be another important indicator of increasing IVDU problems. The risk of developing sepsis increases with repeated exposure to contaminated syringes or untreated open wounds. During IVDU, users may directly introduce harmful bacteria to the bloodstream that may lead to blood, soft tissue, or other internal organ infections. After continued IVDU, other conditions may arise, including osteomyelitis, infective endocarditis, and severe skin and soft tissue infections. Charleston Area Medical Center in Charleston, West Virginia, qualitatively observed increasing rates of sepsis over time.

Research objectives

To investigate whether increased drug use has influenced the rate of sepsis and other infections admissions at Charleston Area Medical Center from 2007-2015.

To investigate whether different types of drug use have increased the odds of developing sepsis compared to skin infections at Charleston Area Medical Center from 2006-2015.

## Materials and methods

Study area

The study patients were all treated at Charleston Area Medical Center. The hospital service area includes the majority of southern West Virginia, parts of eastern Kentucky, and western Virginia.

Data

The Charleston Area Medical Center (CAMC) shared de-identified, Health Insurance Portability and Accountability Act (HIPAA) compliant, digital patient records. These records are created when patients enter the emergency department or hospital. The CAMC Research Division Institutional Review Board (#16-291) provided human subject approval. The first data set analyzed all cases of sepsis treated by the hospital (n=52,735) against all instances of sepsis patients with positive drug use, which include opiates (n=9,873), cocaine (n=402), amphetamines (n=355), sedatives (n=1,359), and mixed drug use defined as two or more drugs being present within a urine test (which is a unique ICD 9/10 code) (n=15,475) from 2006-2015. Table 1 and Table 2 list the ICD 9/10 billing code used to define sepsis and drug use cases. Healthcare providers may routinely order a urine drug test when drug use is suspected. Positive drug cases were identified by urine drug screening, which can detect opioid, cocaine, amphetamine, sedative, and mixed drug use qualitatively. Blood cultures were taken within the emergency department to assess infection within the patient. This study also included state median annual household income provided by the United States Census Bureau (2015). Census data were adjusted and standardized for inflation from 2007-2015.

**Table 1:** ICD 9/10 codes

Drug Codes	ICD 9	ICD 10
Opiates	E850.0, E850.2, 304.00-304.03, 304.70-304.73, 304.50-304.53, 965.01, 965.09	F1120, F1121, F1920, F1921, F1110, T401X1A, T401X2A, T401X3A, T401X4A, T402X1A, T402X2A, T402X3A, T402X4A, T404X1A, T404X2A, T404X3A, T404X4A, T40601A, T40602A, T40603A. T40604A, T40691A, T40692A, T40693A, T40694A
Cocaine	304.21-304.23, 305.60-305.63, 970.81	F1420, F1421, F1410, T405X1A, T405X2A, T405X3A, T405X4A
Amphetamines	304.41-304.43, 305.71-305.73, 969.72	F1520, F1521, F1510, T43621A, T43622A, T43623A, T43624A
Sedatives	305.40-305.43	F1310
Mixed Drug Use/Other	304.60-304.63, 304.80-304.83, 304.90-304.93, 305.90-305.93, 648.33, 648.34	1920, F1921, F1810, O99321, O99322, O99323, O99325

**Table 2:** ICD 9/10 infection codes

Infection Codes	ICD 9	ICD 10
Bacteremia or Sepsis	038.0, 038.10-038.12, 038.19, 038.2, 038.3, 038.40-038.44, 038.49, 038.8, 038.9, 415.12, 422.92, 449, 785.52, 790.7, 995.90-995.92	A409, A412, A4101, A4102, A411, A403, A414, A4150, A413, A4151, A4152, A4153, A4159, A4189, A419, I2690, I400, I76, R6521, R7881, R6510, A419, R6520

The second data set was composed of a sub-analysis of sepsis patients that entered the emergency department and were identified by a positive urine test or blood test as billing code for opiates, cocaine, amphetamines, sedatives, or other illicit substances (n = 2284) within the timeframe of 2007-2015. Patients were further classified as either having or not having the additional categories of sepsis, endocarditis, osteomyelitis, or skin and soft tissue infection. Table 3 summarizes the health outcomes, exposure, and demographic characteristics of the patients, which included age, gender, and race.

**Table 3:** Description of cases

Sex	
Male	1,196
Female	1,088
Race/ethnicity	
White (non-Hispanic)	2,132
Black (non-Hispanic)	131
Other/Unknown	21
Age (years)	
0 – 15	13
16 – 29	553
30 – 39	741
40 – 49	482
50 – 59	359
60 – 69	99
70 – 85	37
Drugs	
Opiates	641
Cocaine	40
Amphetamines	41
Sedatives	151
Mixed Drug Use	1,615
Infections	
Sepsis	995
Skin Infections	1130
Endocarditis	515
Bone Infections	185

Statistical analysis

Equation #1: Infection (Sepsis case count) ^t^ = B1 (Exposure) ^t^ + B2 (Income) ^t^ + B3 (year) ^t^ + e (0, σ 2)

In Equation 1, the dependent variable is the annual sepsis case count and the exposure is the annual count of opiates, cocaine, amphetamines, sedatives, or mixed drug/other usage patients. A separate analysis was conducted for each drug exposure. Income refers to the median West Virginia household income from 2006-2015, which serves as a proxy for increased access to health care [5]. The subscript t refers to years from 2006-2015. The sepsis case count and the number of drug use patients were divided by 100 for ease of interpretation. We verified the time series model assumption of no significant residual autocorrelation using autocorrelation and partial autocorrelation functions of the residuals. The Akaike and Bayesian information criterion selected the best appropriate exposure metric and corresponding time series model. The results section only discusses the statistically significant time series models.

Logistic regression

Equation #2: logit (p) = log (p/ (1-p)) = β0 + β1*Gender + β2*Age + β3*Race + β4*Opiates + β5*Cocaine + β6*Amphetamines + β7*Sedatives+ β8*Mixed

A logistic regression investigated whether specific types of drug use increased the risk of developing sepsis compared to skin infections. The analysis also considered age, gender, and race. The race was categorized as Caucasian (92.1% of cases) and other race (n=152). Logistic regression assumes the linearity of log odds and that observations are independent and identically distributed. The Box Tidwell test for linearity was used to confirm that the final models met this assumption. The analysis also measured model fit using the area under the receiver operating characteristic (ROC) curve. This metric quantifies how often the statistical model correctly classifies those with and without the infection in this case. Separate statistical models compare primary infections (sepsis, endocarditis, and osteomyelitis).

## Results

**Figure 1:**
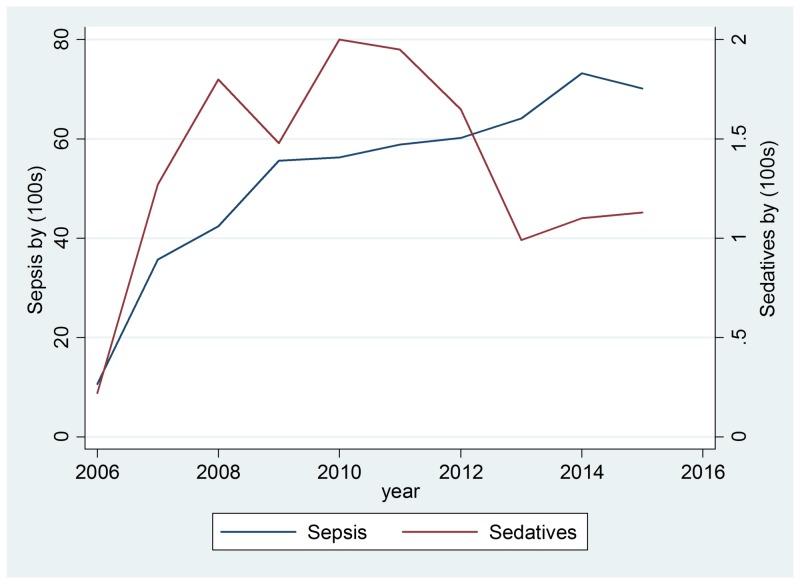
Sepsis cases compared to sedative cases from 2006-2016

**Figure 2:**
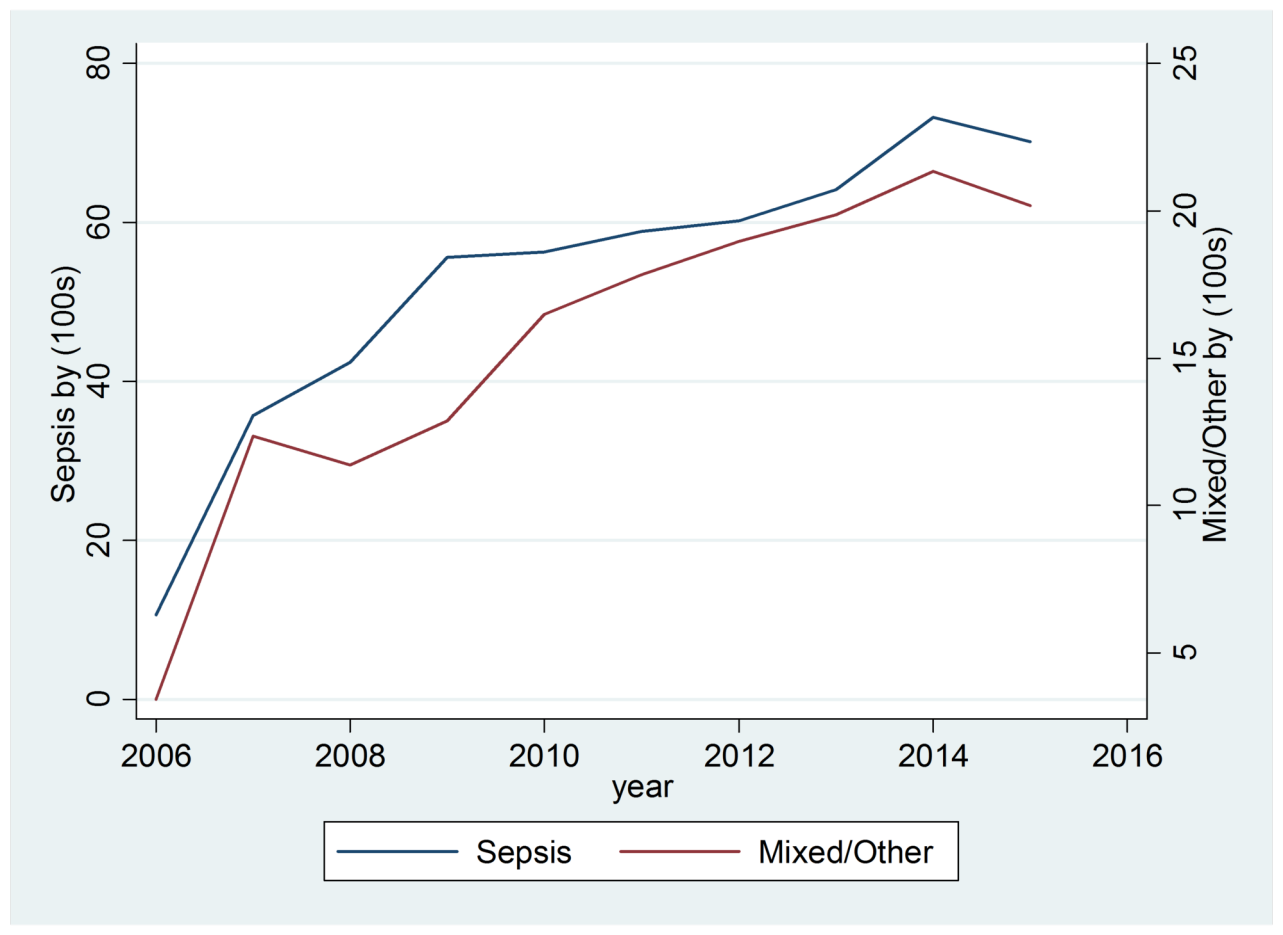
Sepsis cases compared to mixed drug use from 2006-2016

Drug cases with a positive drug test and blood culture are presented in Table 4 and the total cases of drugs and infections are in Table 5. The first analysis investigated the association between drug use and the number of sepsis cases at Charleston Area Medical Center from 2007-2015. Tables 6-7 report the best fitting, statistically significant models that fulfilled the time series assumptions. Results suggest there are similar relationships between sedatives and mixed/other drug use on sepsis cases over time. For every 100 cases of sedative-related drug use entering the hospital, there is an increase of 11.85 sepsis cases, and this relationship was significant (p=0.016). Annual statewide median income did not have a statistically significant relationship with sepsis cases. Figure 1 plots the relationship between observed and time series fitted cases of sepsis and sedative drug use. There is also a statistically significant relationship between sepsis and mixed/other drug use. For every 100 cases of mixed/other drug use, there were 2.8 more sepsis cases (p=0.020). Figure 2 plots the relationship between observed and time series fitted sepsis cases and mixed/other drug exposure.

**Table 4:** Drug cases with positive drug test and blood culture (n = 2284)

Year	Opiates	Cocaine	Amphetamines	Sedatives	Mixed	Sepsis	Skin Infections	Endocarditis	Bone Infection
2007	41	4	0	7	104	54	63	50	7
2008	23	3	0	22	99	52	60	26	11
2009	31	3	0	16	11	70	65	35	7
2010	67	9	2	29	149	104	94	58	14
2011	75	7	5	21	188	117	130	64	18
2012	82	4	3	23	199	116	143	69	16
2013	95	2	6	10	217	127	178	68	24
2014	92	7	8	11	300	172	211	76	43
2015	135	1	17	12	247	183	186	66	45

**Table 5:** Total cases of drugs and infections 2006-2015

Year	Opiates	Cocaine	Amphetamines	Sedatives	Mixed	Sepsis
2006	190	8	0	22	344	1063
2007	793	34	6	127	1237	3564
2008	986	44	9	180	1137	4240
2009	677	35	2	148	1289	5565
2010	917	42	33	200	1649	5629
2011	1055	5	39	195	1783	5885
2012	1039	56	29	165	1896	6025
2013	1177	48	38	99	1988	6414
2014	1248	37	48	110	2134	7324
2015	1791	93	151	113	2018	7026

**Table 6:** Sepsis by sedatives

			R^2^ – Squared = 0.9375 Adj R^2^ – Squared = 0.9063, AIC = 66.14 BIC =67.35	
Sepsis	Beta Coefficient	Standard Error	P>T	95% Confidence Interval
Sedatives	11.85	3.593	0.016	3.061-20.64
Income	.0008	.0036	0.829	-.0081-.0098
Year	5.361	.6343	0.001	3.809-6.913
Cons	-1077	1288	0.001	-1393--7623

**Table 7:** Sepsis by mixed/other

			R^2^ – Squared = 0.9339 Adj R^2^ – Squared = 0.9009 AIC = 66.70 BIC = 67.91	
Sepsis	Beta Coefficient	Standard Error	P>T	95% Confidence Interval
Mixed/Other	2.858	.9053	0.020	.6131-5.073
Income	-.0019	.0037	0.627	-.0111-.0072
Year	.8103	1.647	0.640	-3.221-4.842
Cons	-1540.8	3320	0.659	-9665-6583

For the logistic regression (n=2284), several exposures significantly increased the risk of different infections. Three separate models analyzed the relationship between drug use and severe health outcomes versus skin infections. Of three, tested logistic regression models seen in Tables 8-10, only Table 7 shows the sepsis/skin infection model indicated statistically significant findings for opiates and sedatives. A drug user with a positive urine test for opiates is 80.8% more likely to develop sepsis compared to skin infections (odds ratio of 1.80, 95-confidence interval 1.316-2.486, p=0.001). Sedative usage also significantly increased the odds of developing sepsis by 83.2% (odds ratio 1.832, 95-confidence interval of 1.235-2.718, p = 0.003). These tests were conducted to check differing combinations of emerging infections that might occur in the data set.

**Table 8:** Sepsis/skin infection compared by the following variables logistic regression results ROC: receiver operating characteristic

		N=2284 Area under the ROC curve = 0.6322		
Sepsis	Odds Ratio	Standard Error	P > Z	95 Confidence Interval
Gender	.9331	.0820	0.431	.7853-1.108
Age				
0-15	Comparison			
16-29	.1207	.0940	0.007	.0262-.5558
30-39	.1484	.1154	0.014	.0323-.6817
40-49	.1917	.1494	0.034	.0415-.8835
50-59	.2878	.2249	0.111	.0622-1.331
60-69	.3187	.2553	0.153	.0663-1.531
70-85	.2298	.1935	0.081	.0440-1.197
Race	1.160	.1097	0.116	.9640-1.396
Opiates	1.808	.2936	0.001	1.316-2.486
Cocaine	.7247	.2607	0.371	.3579-1.467
Amphetamines	.6381	.2198	0.192	.3248-1.253
Sedatives	1.832	.3686	0.003	1.235-2.718
Mixed/Other	.9572	.1612	0.795	.6880-1.331

**Table 9:** Endocarditis/skin infection compared by the following variables logistic regression results ROC: receiver operating characteristic

		N=2284 Area under the ROC curve = 0.5615		
Endocarditis	Odds Ratio	Standard Error	P > Z	95 Confidence Interval
Gender	1.298	.1323	0.010	1.063-1.586
Age				
0-15	Comparison			
16-29	3.568	3.745	0.225	.4561-27.91
30-39	4.266	4.473	0.166	.5466-33.30
40-49	4.168	4.378	0.174	.5321-32.65
50-59	4.167	4.383	0.175	.5301-32.75
60-69	4.962	5.300	0.134	.6116-40.26
70-85	4.682	5.183	0.163	.5348-40.99
Race	.7940	.0793	0.021	.6527-.9659
Opiates	1.250	.2301	0.233	.8422-1.794
Cocaine	1.139	.4510	0.741	.5248-2.475
Amphetamines	.7183	.3066	0.439	.3111-1.658
Sedatives	1.219	.2770	0.382	.7815-1.903
Mixed/Other	1.120	.2146	0.553	.7696-1.630

**Table 10:** Osteomyelitis/skin infection compared by the following variables logistic regression results ROC: receiver operating characteristic

		N=2284 Area under the ROC curve = 0.6444		
Osteomyelitis	Odds Ratio	Standard Error	P > Z	95 Confidence Interval
Gender	.8303	.1319	0.242	.6081
Age				
0-15	Comparison			
16-29	1.249	1.301	0.831	.1621-9.628
30-39	2.434	2.504	0.387	.3238-18.29
40-49	4.521	4.650	0.142	.6042-33.94
50-59	3.036	3.144	0.284	.3988-23.11
60-69	1.964	2.166	0.540	.2263-17.05
70-85	NA			
Race	.8284	.1166	0.181	.6286-1.091
Opiates	.7522	.2505	0.393	.3915-1.445
Cocaine	.5202	.4076	0.404	.1120-2.416
Amphetamines	1.864	.9757	0.234	.6685-5.200
Sedatives	.5056	.2291	0.132	.2079-1.229
Mixed/Other	1.115	.3911	0.754	.5613-2.218

## Discussion

Our study suggests that sedatives and mixed/other drug usage have contributed to the increase of Charleston Area Medical Center sepsis cases from 2006-2015. The present study expands the range of health outcomes that are increasing due to amplified drug use in the region [5]. The Rudd study showed the rate of overdoses from 2000-2015 from various states and the concomitant rise in related opioid infections. Overall, opioid overdose deaths have increased from 26 per 100,000 in 2010 in West Virginia to 41 per 100,000 in 2015 [6].

Treating sepsis is one of the largest financial strains on health care institutions [4]. Intravenous (IV) drug users cause a larger financial burden than non-IV drug users and may develop more comorbidities. The current research presented seeks to report further that increasing sepsis rates continue to stress healthcare resources. The average cost of treating a sepsis patient ranges from $22,100-$32,421 USD [1,7-8]. The primary mechanism of IVDU infection is piercing the skin barrier by a non-sterile syringe that may be contaminated by residual bacteria. The syringe transfers bacteria into the bloodstream and may cause an infection leading to sepsis [9-12]. Sepsis can lead to increased health complications and increase the risk of mortality if left untreated [13-17]. The study population is disproportionately suffering from opioid usage due to lower economic status, liberal prescription of opiates to patients, as well as an influx of prescription and illegal drugs into the region [6].

This section describes the limitations of this study. The study relies on case reports and digital data collected by Charleston Area Medical Center. The logistic regression within this analysis showed a similar result to that of the time series results. Opiate and sedative use had a similar relationship. Opiate usage can be explained with the continued increase in the opioid epidemic, but sedative usage also has a similar relationship, which requires further investigation. The data could be slightly over or underreported because of the method of data extraction used, as this study relied on electronic medical records being transcribed and the merger of case files to contrast the analysis. However, considering these limitations, this analysis allows for the construction of a snapshot of the current situation facing this hospital network and its service areas. This study could be improved by using a multi-center analysis as well as a regional analysis to compare differing trends of care and infections. Sepsis, as well as soft tissue infection leading to sepsis, is an easily preventable disease. But because of differing factors that are not limited to the IVDU epidemic, cases are on the rise within this region at an accelerated pace. As the number of opiate users continues to increase, the number of natural sepsis cases will rise as a direct result of other factors, which include the aging demographics of the area and the increase of injuries throughout the region.

## Conclusions

Left untreated, sepsis can be fatal. Our focus should be on prevention. As illicit drug use is most often introduced during adolescent years, reaching out to our youth is essential. Based on current evidence, school-initiated prevention programs, community outreach events, and plans that emphasize parenting skills successfully lower substance abuse rates. Additionally, multiple policies can be created in order to combat this issue, including hospital policies that improve diagnostic techniques, early recognition of sepsis, and appropriate patient education to prevent relapse.
